# Comparison of electrocardiograms (ECG) waveforms and centralized ECG measurements between a simple 6‐lead mobile ECG device and a standard 12‐lead ECG

**DOI:** 10.1111/anec.12872

**Published:** 2021-07-19

**Authors:** Robert Kleiman, Borje Darpo, Randy Brown, Todd Rudo, Svetlana Chamoun, David E. Albert, Johan Martijn Bos, Michael J. Ackerman

**Affiliations:** ^1^ eResearch Technology Inc Philadelphia PA USA; ^2^ PPD Inc Wilmington NC USA; ^3^ AliveCor Corporation San Francisco CA USA; ^4^ Windland Smith Rice Comprehensive Sudden Cardiac Death Program Divisions of Heart Rhythm Services and Pediatric Cardiology Departments of Cardiovascular Medicine, Pediatric and Adolescent Medicine, and Molecular Pharmacology & Experimental Therapeutics Mayo Clinic Rochester MN USA

**Keywords:** Bland–Altman, clinical trials, electrocardiogram, interval duration measurements, QTc, remote monitoring, virtual trials

## Abstract

**Background:**

Interval duration measurements (IDMs) were compared between standard 12‐lead electrocardiograms (ECGs) and 6‐lead ECGs recorded with AliveCor's KardiaMobile 6L, a hand‐held mobile device designed for use by patients at home.

**Methods:**

Electrocardiograms were recorded within, on average, 15 min from 705 patients in Mayo Clinic's Windland Smith Rice Genetic Heart Rhythm Clinic. Interpretable 12‐lead and 6‐lead recordings were available for 685 out of 705 (97%) eligible patients. The most common diagnosis was congenital long QT syndrome (LQTS, 343/685 [50%]), followed by unaffected relatives and patients (146/685 [21%]), and patients with other genetic heart diseases, including hypertrophic cardiomyopathy (36 [5.2%]), arrhythmogenic cardiomyopathy (23 [3.4%]), and idiopathic ventricular fibrillation (14 [2.0%]). IDMs were performed by a central ECG laboratory using lead II with a semi‐automated technique.

**Results:**

Despite differences in patient position (supine for 12‐lead ECGs and sitting for 6‐lead ECGs), mean IDMs were comparable, with mean values for the 12‐lead and 6‐lead ECGs for QTcF, heart rate, PR, and QRS differing by 2.6 ms, −5.5 beats per minute, 1.0 and 1.2 ms, respectively. Despite a modest difference in heart rate, intervals were close enough to allow a detection of clinically meaningful abnormalities.

**Conclusions:**

The 6‐lead hand‐held device is potentially useful for a clinical follow‐up of remote patients, and for a safety follow‐up of patients participating in clinical trials who cannot visit the investigational site. This technology may extend the use of 12‐lead ECG recordings during the current COVID‐19 pandemic as remote patient monitoring becomes more common in virtual or hybrid‐design clinical studies.

## 1 INTRODUCTION

12‐lead electrocardiograms (ECGs) are a standard evaluation included in most clinical trials of new investigational drugs and are used for evaluation of cardiac rhythm, conduction, chamber size, myocardial infarction, potential ischemia, pericarditis, and many other cardiac findings. The interval duration measurements (IDMs), typically including heart rate (HR), PR interval, QRS duration, QT interval, and the heart rate corrected QTc value, are an important part of the evaluation of any ECG. Measurements may be determined by automated ECG machine algorithms, manually measured by the physician/site investigator, or measured by a centralized ECG core laboratory. A major limitation of the standard 12‐lead ECG is related to the placement of the four limb electrodes and the six precordial electrodes. In order to allow reliable measurements and interpretation, as well as to permit comparison between serial ECGs, the electrodes must be placed correctly, with little tolerance for incorrect lead positioning (especially for the precordial leads). The requirements for accurate electrode placement and supine position make it extremely difficult for a patient to record a 12‐lead ECG outside of an investigational site or other medical facilities. It would therefore be very useful to have a method for collecting patient‐recorded ECGs from home that would not require a medical professional with a standard 12‐lead ECG device to visit the patient's home. This unmet need has become highlighted during the current COVID‐19 pandemic, during which many patients enrolled in clinical trials have been unable or unwilling to attend site visits in person. A new device, the AliveCor KardiaMobile 6L, has become available recently for general use, which allows a patient to record a self‐administered 6‐lead ECG, without healthcare professional support, and with minimal instruction (Stavrakis et al., 2017). This study was designed to compare recordings from the AliveCor 6‐lead device to ECGs collected with standard 12‐lead ECG devices.

## 2 METHODS

### 2.1 ECG sources

Patients referred to the Mayo Clinic Windland Smith Rice Genetic Heart Rhythm Clinic between April 2018 and February 2020 were enrolled in a prospective study in which a standard 12‐lead ECG and a 6‐lead mobile ECG were recorded sequentially at the same visit. The 12‐lead ECGs were collected with the patients in the supine position using GE Marquette 12‐lead ECG devices. The 12‐lead ECGs were filtered at 500 Hz, and the digital recordings were stored for analysis. The patients were then allowed to sit up and, after instructions by study nurses, collected a 2‐min recording using the AliveCor KardiaMobile 6L device using both hands and the left leg. Utilizing a smartphone‐based application, the digital files containing the 6‐lead recording were uploaded to a cloud‐based server for subsequent analysis.

### 2.2 6‐lead ECG recordings

**FIGURE 1:**
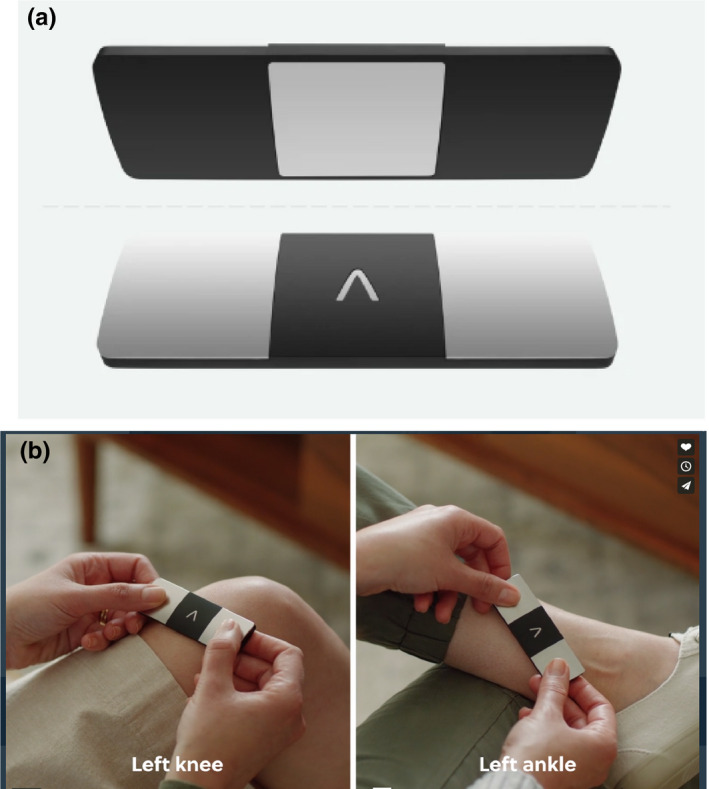
Panel a shows the top and bottom of the AliveCor KardiaMobile 6L. Panel b illustrates a patient positioning the device to record a 6‐lead ECG, with the electrode on the back of the device placed on the left knee or left ankle

The 6‐lead ECGs were recorded with the AliveCor KardiaMobile 6L, a small device (9.0 × 3.0 × 0.72 cm) that has three stainless steel recording electrodes and can thus record the six standard and augmented limb leads. Two electrodes are on the top of the device, while the third is on the bottom (Figure 1a). A patient places the bottom of the device on the left leg (ankle or knee) and touches the top electrodes with fingers from the right and left hands (Figure 1b). This allows recording of standard ECG leads I and II, from which lead III and the augmented limb leads may be derived. ECGs can be recorded from 30 s to 5 min in duration, with a sampling rate of 300 samples/second. The device is connected via Bluetooth to an application loaded into the patient's smartphone, which allows the ECG recordings to be uploaded to AliveCor's Internet cloud‐based servers.

### 2.3 ECG evaluations

Electrocardiograms from each subject were transferred digitally to a centralized ECG core laboratory, eResearch Technology (ERT), and were uploaded into ERT’s validated data management system, EXPERT. IDMs were collected using computer‐assisted caliper placements on three consecutive beats. Trained analysts reviewed all ECGs for correct lead and beat selection, and adjusted the placement of the algorithm placed calipers as necessary using the proprietary validated electronic caliper system applied on a computer screen. A cardiologist then verified the IDMs and performed the morphology analysis.

Electrocardiogram readers were blinded to subject identifiers, treatment, and details of the study. ECGs were evaluated in two separate cohorts (12‐lead ECGs and 6‐lead ECGs) using different subject identifiers and in a randomized order in each cohort, ensuring that readers and cardiologists were not biased by knowledge of how the ECG for the same subject had been measured in the alternate cohort. IDMs for the 12‐lead ECGs were performed on the unfiltered Lead II whenever possible. When Lead II was not analyzable, the secondary measurement lead was V5, and the tertiary measurement lead was V2. IDMs from 6‐lead ECGs were performed on lead II after filtering. When Lead II was not analyzable, the secondary measurement lead was Lead I, and the tertiary measurement lead was Lead III. Mean values were generated from the individual ECG measurements; at the beat level, QT was corrected based on the preceding RR interval, generating a beat‐level QTc. The mean of the three beat‐level QTc values was then reported as the mean QTc value for the ECG.

### 2.4 Statistical methods

The Bland–Altman method was used as the primary comparison method (Bland & Altman, 1986, 1995). Mean IDMs from each subject's 12‐lead ECG were subtracted from the values obtained from the 6‐lead ECG, and differences were displayed as a function of the mean of the two measurements. Limits of agreement (LoA) and 2‐sided 95% confidence intervals (CI) for the mean difference and LoA were calculated.

Bias analysis was also performed to assess the potential bias of measurements between recording devices. Relationship between the means and differences of the ECG intervals between the two methods was assessed using robust regression using an M estimator (Ferber et al., 2017). The fitted slope (“BA slope”) and associated 2‐sided 95% CIs are derived to show the linear trends between the two variables. The BA slope indicates whether the observed difference between methods varies with the magnitude of the absolute value.

## 3 RESULTS

Interpretable 12‐lead and 6‐lead recordings were available for 685 out of 705 (97%) eligible patients enrolled prospectively between April 2018 and February 2020. The average patient age was 28.7 ± 18.5 years, with 43% males and 57% females. The most common diagnosis was LQTS (343/685 [50%]), followed by unaffected relatives and patients who were determined to be normal after a comprehensive cardiovascular evaluation (146/685 [21%]). Smaller numbers of patients had other genetic heart diseases, including hypertrophic cardiomyopathy (36 [5.2%]), arrhythmogenic cardiomyopathy (23 [3.4%]), and idiopathic ventricular fibrillation (14 [2.0%]).

Eleven subjects (1.6%) had more than 30‐min elapse between the 12‐lead and 6‐lead recordings; these data were not included in the data analysis due to the long interval between recordings. For the remaining 674 subjects, the mean interval between the 12‐lead and 6‐lead ECG recordings was 12.4 min (standard deviation: 4.1 min).

Overall, patients found the AliveCor KardiaMobile 6L device easy to use and reported no difficulties with using it for recording ECGs.

### 3.1 ECG recording quality

**FIGURE 2:**
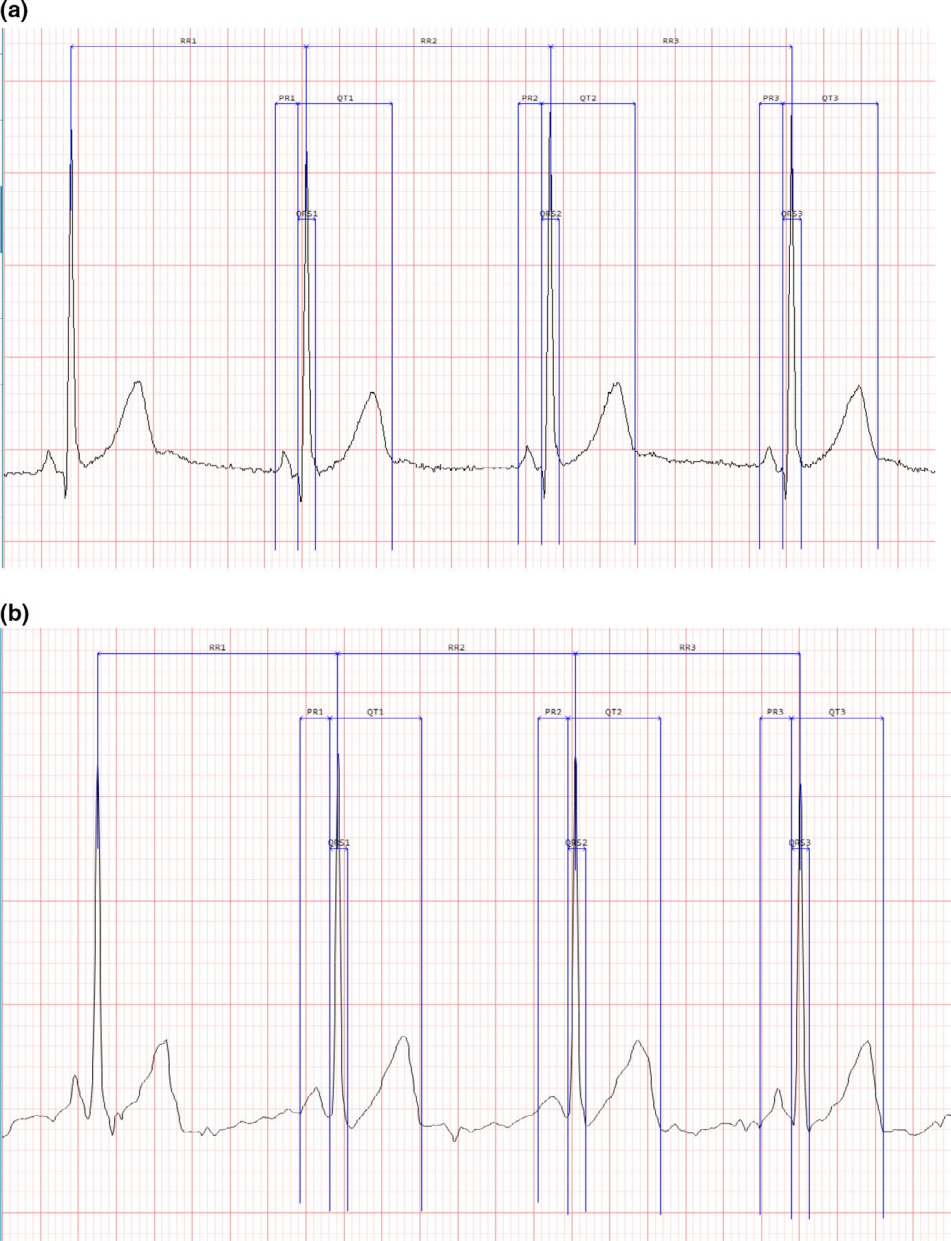
Lead II waveforms with annotated caliper placements from 12‐lead (panel a, unfiltered) and 6‐lead (panel b, with filtering) ECGs recorded from the same subject

A comparison of the recording quality between the paired 12‐lead and 6‐lead recordings revealed that in general, both were of good quality. All 12‐lead ECGs were of sufficient quality to allow IDM measurements and cardiologist interpretation, and only one of the 6‐lead ECGs was unsuitable for IDM measurements (due to excessive artifact), though adequate for cardiologist interpretation. The unfiltered 12‐lead ECGs had less artifact and did not require filtering prior to performing measurements, while the unfiltered 6‐lead ECGs had significantly more artifact and required filtering before IDMs could be performed. This resulted in small differences in the ECG waveform morphology that were apparent only at high magnification. Figure 2 illustrates the lead II waveforms with caliper annotations for the 12‐lead and 6‐lead recordings of a representative subject.

### 3.2 ECG waveform morphology

**FIGURE 3:**
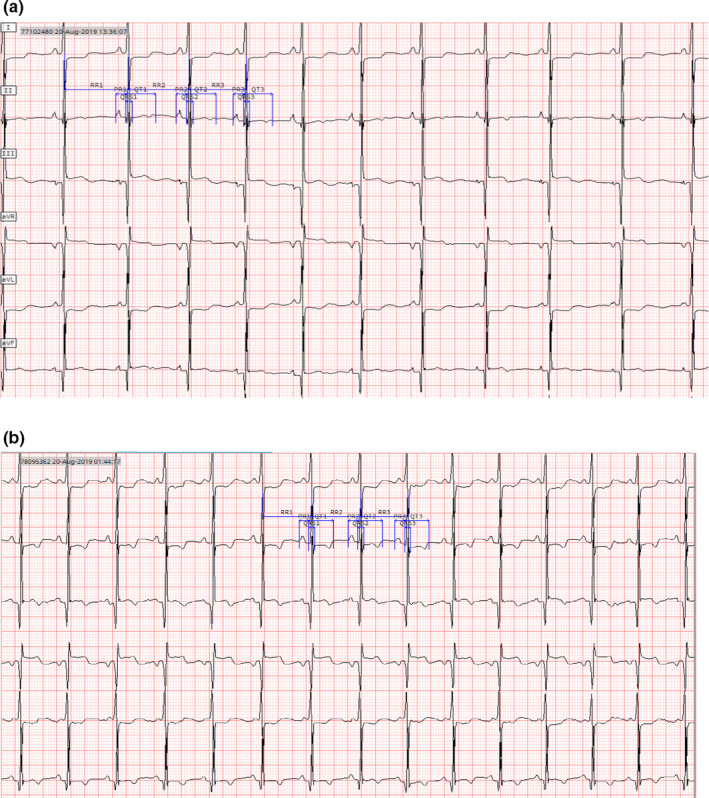
12‐lead (panel a) and 6‐lead (panel b) ECGs from the same subject with more prominent ST and T‐wave findings on the 6‐lead recording

The 12‐lead and 6‐lead ECGs were similar in morphology (limb leads), although there were some subjects for whom there were substantial differences in ECG morphology between the two recordings. As examples, some pairs of ECGs showed ventricular pacing in one recording, and a non‐paced rhythm in the other, or had T‐wave inversion in one recording but not the other. Since the ECGs were recorded with the subjects in different positions (supine versus sitting) and with an interval of 5–30 min between them, it is likely that these morphologic changes represent true ECG morphology changes rather than being related to the recording method. Figure 3 illustrates a pair of ECGs from a subject that have differences in the appearance of the ST segments and T waves.

### 3.3 Interval duration measurements

A summary of the mean values for the IDMs with the mobile 6‐lead and standard 12‐lead recordings is shown in Table 1. Fifty of the 6‐lead recordings and fifty‐one of the 12‐lead recordings could not be measured in lead II and were measured in a secondary lead.

**TABLE 1:** Observed values for 6‐lead and 12‐lead ECG measurements and differences with descriptive statistics

Parameter	Statistic	KM 6‐lead ECG	12‐lead ECG	Difference (KM 6L–12L)
QTcF (ms)	*N*	671	674	671
	Mean (SD)	428.5 (36.50)	431.0 (38.80)	−2.6 (19.81)
	95% Confidence interval	425.73; 431.27	428.11; 433.97	−4.06; −1.06
	Median	427.0	427.0	−2.0
	Min/Max	327/746	316/744	−125/68
HR (bpm)	*N*	674	674	674
	Mean (SD)	71.9 (14.59)	66.5 (14.24)	5.4 (7.51)
	95% Confidence interval	70.84; 73.05	65.42; 67.57	4.88; 6.02
	Median	71.0	65.0	5.0
	Min/Max	41/121	38/121	−27/37
PR (ms)	*N*	672	664	663
	Mean (SD)	154.3 (24.95)	155.2 (25.18)	−1.0 (14.69)
	95% Confidence interval	152.45; 156.23	153.24; 157.08	−2.09; 0.15
	Median	152.0	153.0	0.0
	Min/Max	96/269	83/272	−72/62
QRS (ms)	*N*	673	674	673
	Mean (SD)	93.1 (11.96)	91.9 (12.89)	1.2 (9.11)
	95% Confidence interval	92.15; 93.96	90.90; 92.85	0.48; 1.86
	Median	92.0	90.0	2.0
	Min/Max	72/174	70/185	−46/30
QT (ms)	*N*	671	674	671
	Mean (SD)	407.5 (49.14)	420.9 (51.87)	−13.5 (20.83)
	95% Confidence interval	403.73; 411.17	416.97; 424.82	−15.09; −11.93
	Median	405.0	419.0	−12.0
	Min/Max	290/792	306/791	−138/47

Abbreviations: bpm, beats per minute; HR, heart rate; Max, maximum; Min, minimum; ms, milliseconds; SD, standard deviation.

### 3.4 Heart rate

**FIGURE 4:**
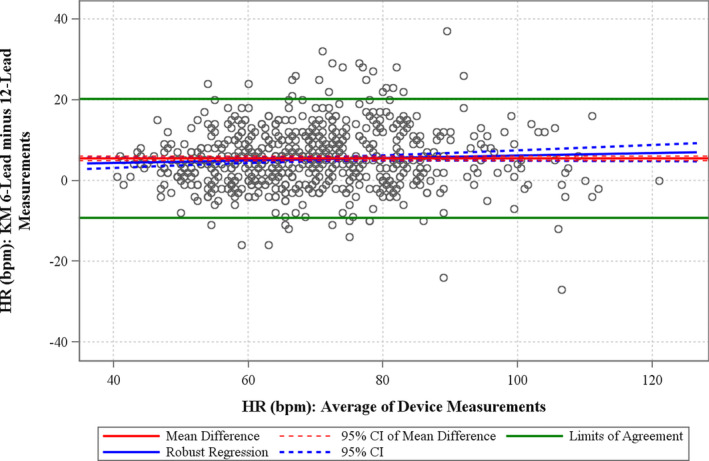
Bland–Altman and bias assessment plots for heart rate (HR). The solid horizontal red line represents the mean difference, and the hashed red line represents the 95% confidence bounds for the measurement pairs. The horizontal green lines represent the limits of agreement

The Bland–Altman and bias analysis plots for heart rate are shown in Figure 4. The horizontal green lines represent the LoA; this represents the range in which the difference between two measurements is expected for 95% of future measurement pairs. The mean difference between the HR as measured on the 6‐lead and 12‐lead ECGs was 5.5 beats per minute (bpm; 95% confidence interval [CI] 4.9–6.0 bpm). The 12‐lead ECGs were recorded while the subjects were supine, and the 6‐lead ECGs while the patients were sitting up and applying the AliveCor ECG device themselves; this likely is the explanation for the higher HR for the 6‐lead ECGs.

The bias analysis for HR demonstrated a BA slope of 0.030 (95% CI −0.008; 0.069), meaning that the mean difference between methods was 0.3 bpm over a HR range of 10 bpm.

### 3.5 QTcF

**FIGURE 5:**
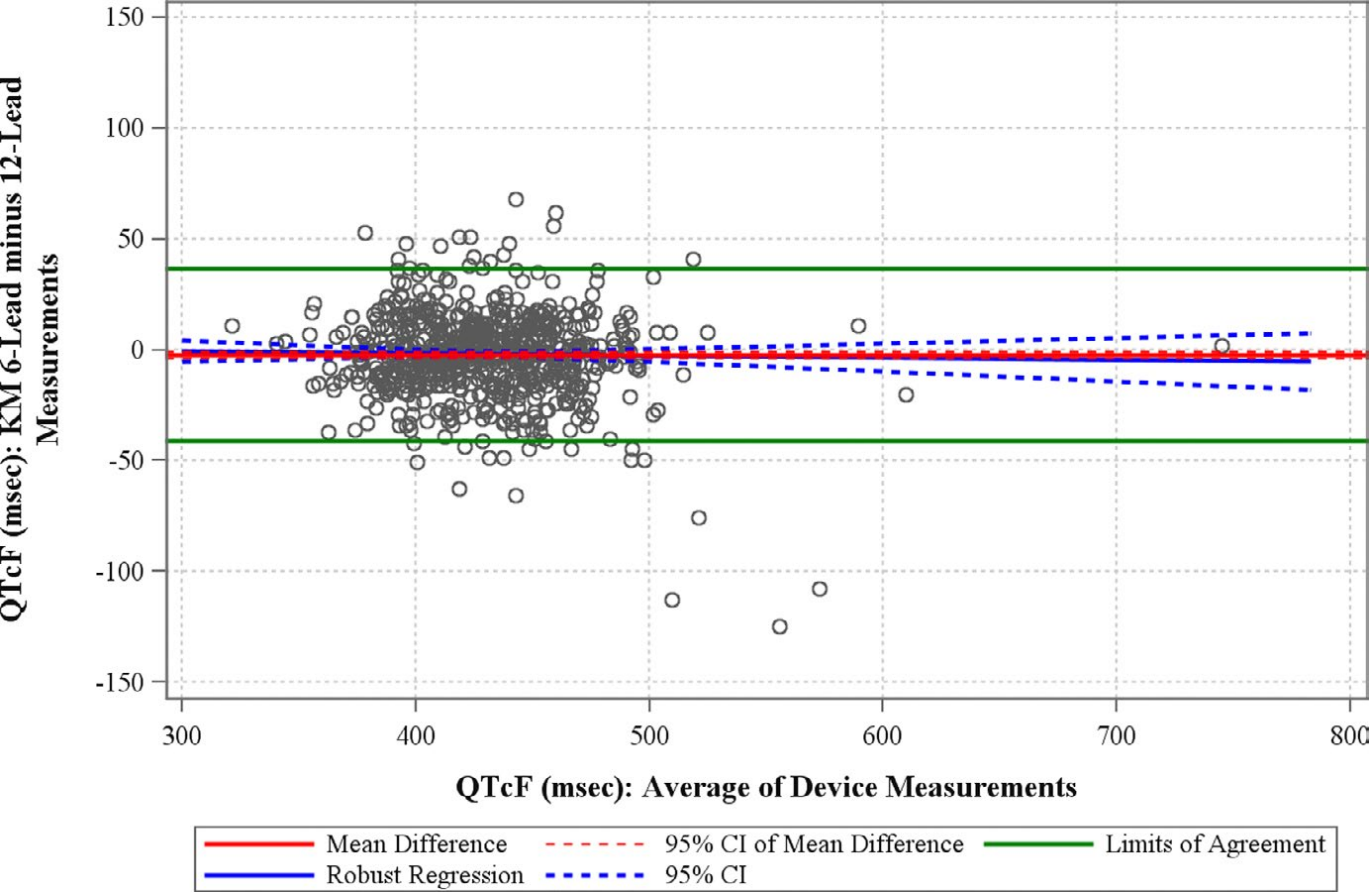
Bland–Altman and bias assessment plots for QTcF. The solid horizontal red line represents the mean difference, and the hashed red line represents the 95% confidence bounds for the measurement pairs. The horizontal green lines represent the limits of agreement

The Bland–Altman plot for QTcF is shown in Figure 5. The mean difference between the QTcF measured on the 6‐lead and 12‐lead ECGs was −2.6 ms (95% CI −4.1; −1.1 ms). The BA slope was −0.010 (95% CI −0.046; 0.026), that is, indicating a mean difference between methods of 1 ms over a QTcF range of 100 ms.

**FIGURE 6:**
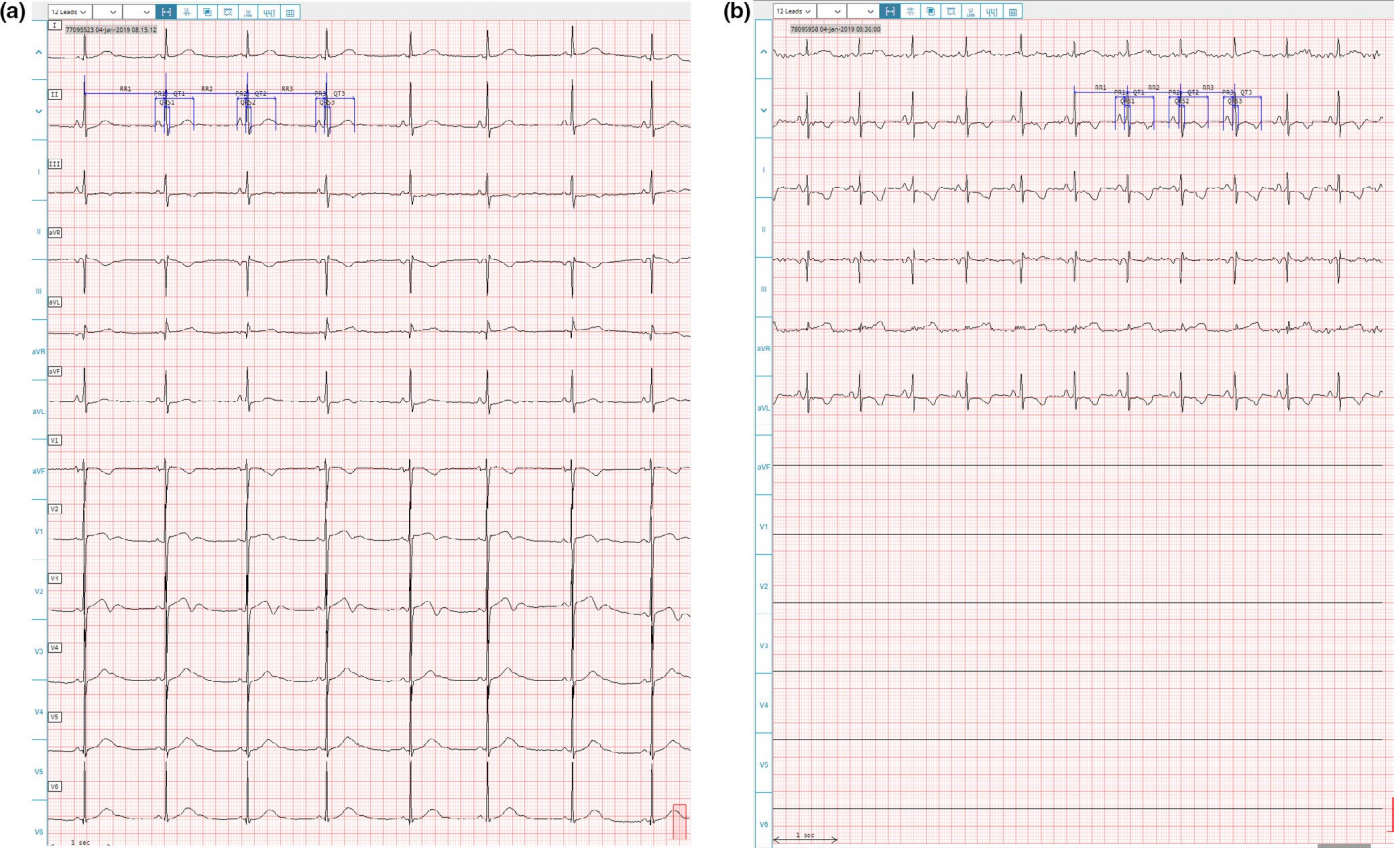
12‐lead (panel a) and 6‐lead (panel b) ECGs for subject with a 62 ms difference between QTcF measurements. The 12‐lead ECG was recorded while the T waves in the measurement lead were upright, while the 6‐lead ECG recorded 24 min later had inverted T waves in the measurement lead

There were patients who had large (>50 ms) differences in QTcF, which represented true changes in the ECG between the two recordings. The largest difference in QTcF measurements was −125 ms; the 12‐lead ECG was recorded during a period of ventricular pacing, while the 6‐lead ECG was recorded at a time when the QRS complexes were not paced, resulting in large differences between the QT (and QRS) measurements. An example of the 12‐lead and 6‐lead ECGs from a patient who had a 62 ms difference in QTcF due to significant changes in T‐wave morphology is shown in Figure 6.

### 3.6 PR interval

**FIGURE 7:**
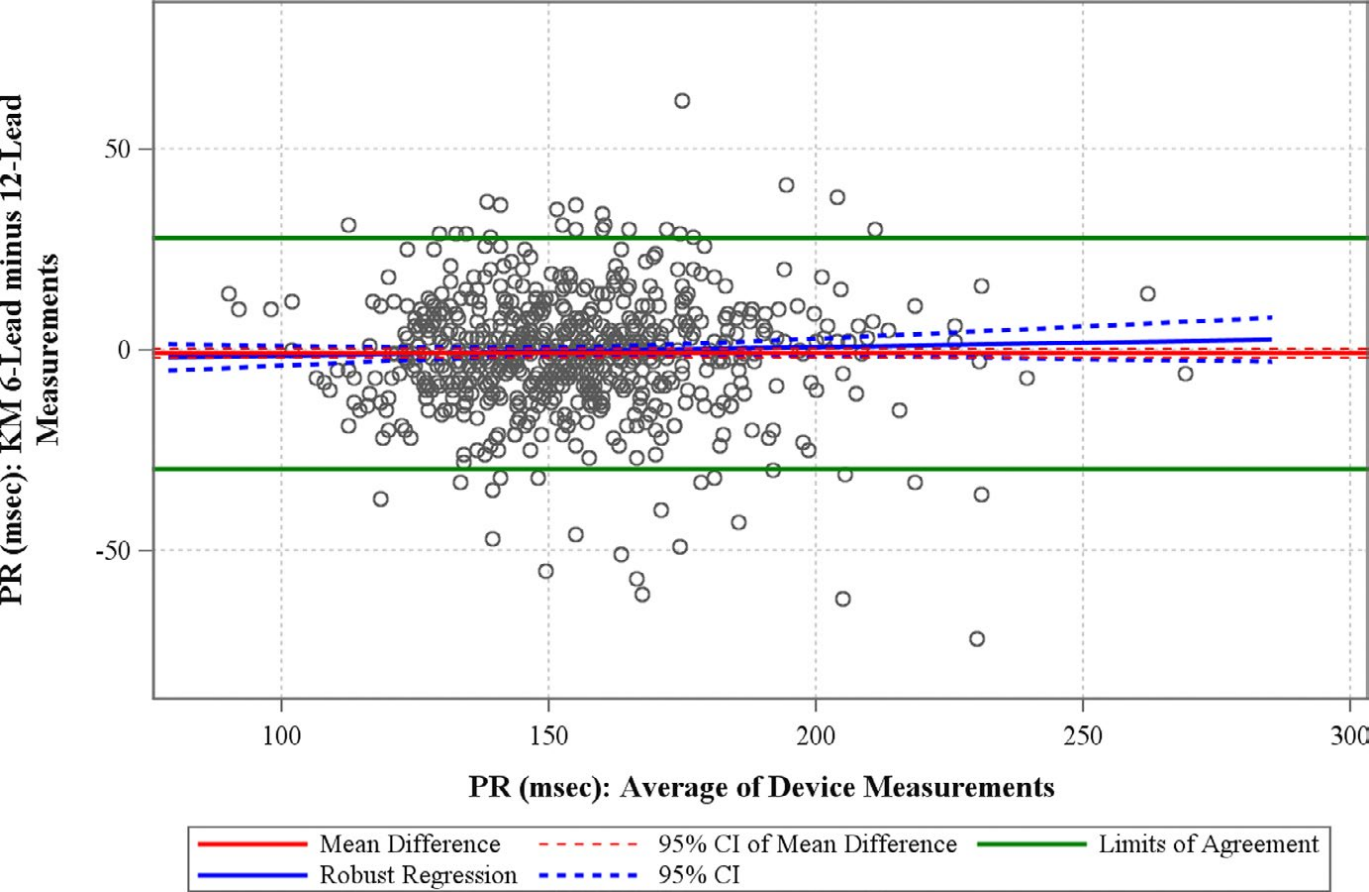
Bland–Altman and bias assessment plots for the PR interval. The solid horizontal red line represents the mean difference, and the hashed red line represents the 95% confidence bounds for the measurement pairs. The horizontal green lines represent the limits of agreement

The Bland–Altman and bias assessment plots for PR are shown in Figure 7. The mean difference between the PR interval measured on the 6‐lead and 12‐lead ECGs was −0.97 ms (95% CI −2.086; 0.155 ms). The bias analysis demonstrated a BA slope of 0.021 (95% CI −0.0196; 0.0626), which means that the mean difference between methods was 2.1 ms over a PR range of 100 ms.

### 3.7 QRS duration

**FIGURE 8:**
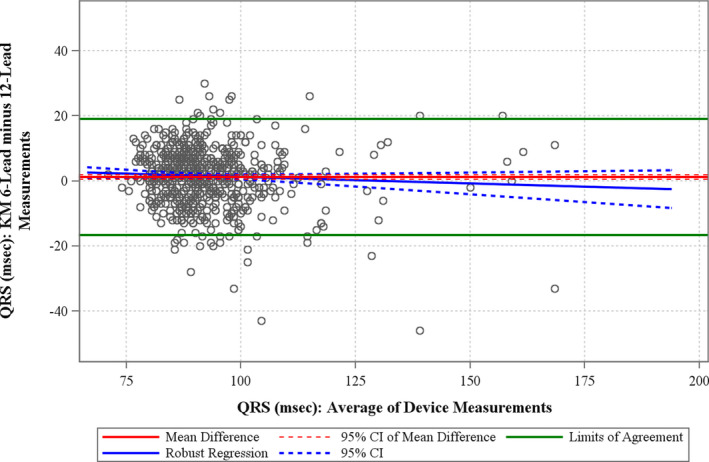
Bland–Altman and bias assessment plots for QRS. The solid horizontal red line represent the mean difference, and the hashed red line represents the 95% confidence bounds for the measurement pairs. The horizontal green lines represent the limits of agreement

The Bland–Altman and bias assessment plots for QRS duration are shown in Figure 8. The mean difference between the QRS duration measured on the 6‐lead and 12‐lead ECGs was 1.17 ms (95% CI 17.843; 20.195 ms). The bias analysis demonstrated a BA slope of −0.041 (95% CI −0.0969; 0.0167), that is, the mean difference between methods was 0.4 ms over a QRS range of 10 ms.

A summary of the agreement for the interval duration measurements of the Bias Assessment is presented in Table 2. The results of categorical analyses of differences in ECG intervals between the measurements derived from the 6‐lead recordings and the 12‐lead recordings are presented in Table 3.

**TABLE 2:** Summary of differences between interval duration measurements from 6‐lead and 12‐lead ECGs with upper and lower limits of agreement and bias sensitivity results

Parameter	Difference: 6‐lead minus 12‐lead (95% CI)	Lower limit of agreement	Upper limit of agreement	BA slope	Standard error	95% CI
QTcF (ms)	−2.6 (−4.1; −1.1)	−41.39	36.26	−0.01	0.018	−0.05; 0.03
HR (bpm)	5.5 (4.9; 6.0)	−9.28	20.17	0.03	0.02	−0.01; 0.07
PR (ms)	−1.0 (−2.1; 0.2)	−29.76	27.83	0.02	0.02	−0.02; 0.06
QRS (ms)	1.2 (0.5; 1.9)	−16.67	19.02	−0.04	0.03	−0.10; 0.02

Abbreviations: BA, Bland–Altman; bpm, beats per minute; CI, confidence intervals; HR, heart rate; ms, milliseconds.

**TABLE 3:** Categorical analysis of difference in ECG intervals between 6‐lead and 12‐lead recordings

Parameter	Category	Frequency (percent %)
QTcF ms (*n* = 671)	Absolute difference <10	297 (44.3%)
	10 ≤ Absolute difference <20	221 (32.9%)
	20 ≤ Absolute difference <30	69 (10.3%)
	30 ≤ Absolute difference <40	50 (7.5%)
	40 ≤ Absolute difference <50	19 (2.8%)
	Absolute difference ≥50	15 (2.2%)
HR bpm (*n* = 674)	Absolute difference <10	478 (70.9%)
	10 ≤ Absolute difference <20	168 (24.9%)
	20 ≤ Absolute difference <30	26 (3.9%)
	30 ≤ Absolute difference <40	2 (0.3%)
	40 ≤ Absolute difference <50	0
	Absolute difference ≥50	0
PR ms (*n* = 663)	Absolute difference <10	383 (57.8%)
	10 ≤ Absolute difference <20	184 (27.8%)
	20 ≤ Absolute difference <30	58 (8.7%)
	30 ≤ Absolute difference <40	25 (3.8%)
	40 ≤ Absolute difference <50	6 (0.9%)
	Absolute difference ≥50	7 (1.1%)
QRS ms (*n* = 673)	Absolute difference <10	492 (73.1%)
	10 ≤ Absolute difference <20	158 (23.5%)
	20 ≤ Absolute difference <30	18 (2.7%)
	30 ≤ Absolute difference <40	3 (0.4%)
	40 ≤ Absolute difference <50	2 (0.3%)
	Absolute difference ≥50	0

Abbreviations: bpm, beats per minute; ms, milliseconds.

## 4 DISCUSSION

The 12‐lead ECG is an important tool that is used regularly in clinical practice and in nearly all clinical trials. The complexity of placing the limb, and especially precordial leads, has traditionally limited the collection of 12‐lead ECGs to physician offices, hospitals, and diagnostic laboratories. While it is possible for a healthcare professional to bring a 12‐lead ECG machine to a patient's home to record an ECG, this is an expensive and complex undertaking and is rarely performed. It would be extremely helpful, especially during the current COVID‐19 pandemic, to be able to collect high‐quality self‐administered ECGs at home, with the patient capable of recording and transmitting the ECG to the patient's physician (and to the ECG core laboratory during a clinical trial). This would also be a very useful extension to the standard use of office/hospital‐based 12‐lead ECGs and would enable the collection of ECGs either whenever a patient has cardiac symptoms while at home, or as part of a clinical trial but without requiring a visit to the investigational site.

The AliveCor KardiaMobile 6L is a simple, mobile device that allows almost any patient to collect a 6‐lead ECG comprised of the standard limb leads easily and quickly, and then transmit the ECG for evaluation. While a 6‐lead ECG is not a replacement for a 12‐lead ECG in all situations, a full set of limb lead recordings is perfectly adequate for assessment of cardiac rate and rhythm, AV conduction, as well as the standard IDMs (RR, PR, QRS, QT/QTc). By contrast, in the absence of precordial leads, detection of other diagnoses is far more limited, such as detection of anterior wall ischemia or infarction, or repolarization syndromes manifest primarily in the chest leads. The standard IDMs are normally measured in lead II, and the AliveCor 6‐lead device is therefore a useful tool for allowing QTc assessments without the need for the patient to visit a medical facility—thus potentially expanding our ability to follow QTc measurements over time remotely as a patient's medical condition or prescribed medications evolve.

During the current study, we have compared the IDMs collected with a standard 12‐lead ECG and the AliveCor 6‐lead device in a population comprised not of normal healthy subjects, but instead in a population followed in a genetic heart rhythm clinic who had markedly abnormal ECGs. Although the ECGs were not recorded simultaneously, the mean values for the IDMs were remarkably consistent, other than the small difference in measured heart rate, which was likely related to the change in patient position from supine to sitting. The Bland–Altman slopes for the IDMs demonstrated no evidence of systematic measurement bias at high or low measurement values.

In any individual subject, HR, PR, QRS, and QTc vary somewhat over intervals of as short as a few minutes. This variability is exacerbated by any event that would produce a physiologic change (such as a change in position, the addition of a drug that prolongs PR or QTc, ischemia, or an intermittent bundle branch or pacing; Dilaveris et al., 2001; Molnar et al., 1996) In particular, QTc has very wide variability over time (partly due to changes in HR or autonomic tone; Morganroth et al., 1991) Since the ECGs in this study were not collected simultaneously (often with 10–20 min between recordings) or with the patient in the same position, the two QTcF measurements for a single patient would not be expected to be identical. Due to changes in position between the two recordings, the mean HR change was >10 bpm in 29% of patients. Nevertheless, the mean differences in QTcF and the other intervals were quite small, despite the differences in patient position, changes in HR, and the time interval between the two recordings. In a large enough population, one would expect that in the absence of any significant physiologic events, repeat ECG measurements recorded 5–30 min apart would demonstrate very small mean changes, whether the recordings were performed with the same or different ECG devices. The results of this study confirm that ECG measurements remain, on average, relatively stable over short intervals. A few instances of large differences in intervals were observed, and in reach, instances were related to significant changes in ECG rhythm or T‐wave morphology.

The results of this study suggest that the use of this smartphone‐enabled, mobile technology would be appropriate for many uses in clinical medicine and during clinical trials. This is not the device that one would want to use for patients with unstable angina, but would be ideal to allow following the rhythm of a patient who has had paroxysmal atrial fibrillation or an atrial fibrillation ablation, or for following the QTc value of a patient receiving one or more QT‐prolonging medications. This would enhance our ability to assess patient safety between scheduled visits (rapid assessment of new symptoms or period checks to detect large increases in QTc, PR, or QRS).

### 4.1 Limitations

The two primary limitations of this study were that the two sets of ECGs were not recorded (i) simultaneously or (ii) with the patient in the same position. Due to the variability of IDMs with time, as well as changes in autonomic tone and heart rate with position, it was not expected that the measurements taken 5–15 min apart would be identical. This limits the utility of these data for comparing the precision of the ECG measurements made with the two ECG recording methods. However, the mean differences in QTcF, PR, and QRS were all very small, likely because the variability of these measurements was evenly distributed across the patients. Thus, the data do not indicate a consistent, systemic bias when comparing the two methodologies. For some patients, QTcF increased, but for a similar number, QTcF decreased. The increase in HR is to be expected due to the change from a supine position (12‐lead ECG) to sitting (6‐lead ECG). The order of assessments was also fixed (12‐lead ECGs recorded first, followed by training and the recording of 6‐lead ECGs), though this was unlikely to have had a significant effect on the results. The secondary lead used for measurements when lead II was not adequate for precise measurements was different for the two data types (lead V5 was generally the secondary lead for the 12‐lead ECGs, while lead I was generally the secondary lead for the 6‐lead ECGs). The contribution of lead changes to the IDM differences is uncertain.

Another potential limitation is that this study was conducted among patients participating in a genetic heart rhythm clinic at a referral institution and therefore may not adequately represent the results that would be observed in the general population. The average age of this population was 28.7 years, which is lower than the average age of the US population (38.5 years). However, since many of these patients had QTc prolongation due to congenital structural cardiac or rhythm abnormalities, this population included primarily patients with abnormal ECGs and, compared with the general population, may represent the “worst case” for ECG measurements.

## 5 CONCLUSIONS

In conclusion, this study has demonstrated that 6‐lead recordings of the limb leads using a novel but simple‐to‐use smartphone‐enabled, mobile device can provide high‐quality ECG recordings that may be useful for many purposes in clinical medicine and during clinical trials. This technology should not be viewed as a replacement for 12‐lead ECGs, for it is not. Instead, it may represent a valuable method for expanding our reach for collecting high‐quality ECG data by remotely acquiring patient‐administered 6‐lead ECGs. This may be immensely valuable during the current pandemic, during which many patients are reluctant to visit their local physician's office, in virtual or hybrid‐design clinical studies, as well as for general use to expand our ability to collect ECG recordings when a patient is not at a medical facility.

## CONFLICTS OF INTEREST

RK, TR, and SC are employees of ERT, a company that offers centralized ECG reading services to the biopharmaceutical industry. BD is a consultant for ERT and owns stock and is eligible for stock options with the company. MJA and Mayo Clinic have a potential equity/royalty relationship with AliveCor.

## AUTHOR CONTRIBUTIONS

MJA and JMB organized collection of the ECGs at the Mayo Clinic Windland Smith Rice Genetic Heart Rhythm Clinic; RB performed the statistical analyses; RK wrote the first draft of the manuscript, and all authors participated in revision and approval of the submitted version.

## ETHICAL APPROVAL

The prospective ECG collection for this study was approved by the Mayo Clinic Instutional Review Board.

## Data Availability

The data that support the findings of this study are available on request from the corresponding author. The data are not publicly available due to privacy or ethical restrictions.
